# A dual-path convolutional neural network combined with an attention-based bidirectional long short-term memory network for stock price prediction

**DOI:** 10.1371/journal.pone.0319775

**Published:** 2025-04-22

**Authors:** Guiyan Zhao, Yunfei Cheng, Jianhui Yang, Jiayuan Ouyang

**Affiliations:** 1 Qiaoxing School of Economics and Management, Fujian Polytechnic Normal University, Fuzhou, China; 2 School of Mathematics and Statistics, Changchun University of Technology, Changchun, China; Air Force Engineering University, CHINA

## Abstract

The complexities of stock price data, characterized by its nonlinearity, non-stationarity, and intricate spatiotemporal patterns, make accurate prediction a substantial challenge. To address this, we propose the DCA-BiLSTM model, which combines dual-path convolutional neural networks with an attention mechanism (DCA) and bidirectional long short-term memory networks (BiLSTM). This model captures deep information and complex dependencies within time-series data. First, wavelet packet decomposition extracts high- and low-frequency features, followed by DCA for robust deep feature extraction, and finally, BiLSTM models bidirectional dependencies. Validated on datasets from Yahoo Finance, including Apple, Google, Tesla stocks, and the Nasdaq index, the model consistently outperforms traditional approaches. The DCA-BiLSTM achieves an $R^{2}$ of 0.9507 for Apple, 0.9595 for Google, 0.9077 for Tesla, and 0.9594 for the Nasdaq index, with significant reductions in error metrics across all datasets. These results demonstrate the model’s robustness and improved predictive accuracy, offering reliable insights for stock price forecasting.

## 1 Introduction

The financial market, as a complex macro system, is influenced by various macroeconomic, political, and social factors. Stock price fluctuations are widely regarded as effective indicators of overall economic health, and changes in the stock market significantly impact economic activities and corporate development. Consequently, researchers have long sought to develop effective models for predicting stock prices [1,2]. However, stock price prediction is inherently complex due to the multitude of factors affecting prices, which renders traditional models inadequate for capturing dynamic market patterns [3]. With advancements in big data processing technologies and AI algorithms, data science methodologies have achieved notable success in the financial domain. Accordingly, research leveraging diverse AI algorithms is expected to become a vital tool for financial researchers analyzing market dynamics, particularly in overcoming the limitations of traditional approaches.

Stock price prediction often involves intricate nonlinear relationships that traditional linear models struggle to capture effectively. Multiple factors, including company fundamentals, technical indicators, and market sentiment, influence price movements. AI algorithms, with their ability to process vast amounts of data and uncover latent patterns, provide a flexible and powerful solution. Unlike traditional economic models bound by rigid assumptions, these algorithms learn from historical data without constraints, adapting to complex data patterns. This adaptability is particularly valuable for real-time decision-making in financial trading.

In recent years, deep learning-based models for stock prediction have emerged as a focal area of research, with specific emphasis on convolutional neural networks [4–6], recurrent neural networks [7,8], and Transformers [9,10]. Hybrid approaches have garnered considerable attention, aiming to combine the strengths of multiple models to achieve higher predictive accuracy compared to traditional single-model approaches. Jaiswal and Singh introduced a novel hybrid convolutional-recurrent model that integrates a one-dimensional convolutional neural network (CNN) with a gated recurrent unit (GRU) model. In this model, CNN is used for feature extraction, while GRU manages temporal regression, showing superior performance over traditional models [11]. Rostamian and Hara proposed a model that incorporates long short-term memory (LSTM) with CNN, effectively combining LSTM’s robustness in temporal prediction with CNN’s strengths in feature extraction, leading to significant improvements over conventional models [12]. Nevertheless, despite the advancements offered by these hybrid models [13], there is still room for improvement. Some research advocates for the use of bidirectional recurrent neural network (RNN) models [14,15] and the integration of attention mechanisms [16] to further enhance feature extraction. Against this background, Lu et al. and Luo et al. presented attention-based models that adopt a CNN-BiLSTM fusion structure, where CNN is utilized for feature extraction and BiLSTM for regression prediction [17,18]. By incorporating attention mechanisms, these models effectively capture the influence of different temporal feature states on stock closing prices, thereby improving prediction accuracy. Wang proposed an alternative approach involving a temporal convolutional network that combines BiLSTM with enhanced Transformers, leveraging Transformers to capture global information and BiLSTM to learn bidirectional short-sequence information. Experimental results reveal its significant advantages over standalone Transformer models [19]. In summary, combining multiple models for prediction research offers distinct advantages over single-model approaches.

In stock prediction tasks, studies suggest that dual-channel or even multi-channel structures can significantly enhance model performance by performing deeper analyses of data features [20–25]. To further improve the effectiveness of predictive models, this paper proposes an enhanced approach based on the traditional hybrid model. This model incorporates a neural network that integrates dual-path convolutional attention mechanisms with BiLSTM. Furthermore, to enhance the dual-path CNN’s ability to extract multi-scale features, we employ a wavelet packet coefficient decomposition method for dataset preprocessing. This allows the dual-channel CNN, operating at multiple scales, to extract more comprehensive features by separating high and low-frequency data through filtering. However, due to the relatively complex multi-scale feature structures extracted by the dual-channel CNN, directly using BiLSTM for regression poses challenges for datasets with longer periods. Therefore, we introduce an improved multi-scale feature fusion approach leveraging attention mechanisms to enhance performance on stock price datasets.

The structure of this paper is as follows: Section 2 provides a detailed overview of the proposed DCA-BiLSTM stock prediction model. Section 3 discusses the experimental validation and results. Section 4 concludes the paper, summarizing key findings.

## 2 Stock price prediction model

Given the nonlinearity, non-stationarity, and complex spatiotemporal relationships inherent in stock price data, this study introduces a stock price prediction model based on DCA-BiLSTM. The goal is to improve prediction accuracy by extracting and effectively leveraging highly correlated and diverse deep features during the regression process.

This study examines stock price prediction under a scenario involving *n* observation samples. For each target date *i*, the closing price $y_{i}$ on day *i* is treated as the dependent variable in regression analysis. The independent variable data $x_{i}$ includes metrics such as opening price, highest price, lowest price, price change, among others. By combining the independent variable data $x_{i}$ with the dependent variable data $y_{i}$, we form the observation dataset ${\left\{y_{i},x_{i}\right\}}_{i=1}^{n}$.

The stock price prediction model proposed in this paper consists of three main stages:

(1) Decomposing the raw input time series data $x_{i}$ into high-frequency domain signals $x_{j,u}^{2m}$ and low-frequency domain signals $x_{j,u}^{2m+1}$ using wavelet packet decomposition, and transforming them into shallow high-low frequency domain features through a feature extraction layer;
(2) Using a dual-path convolutional neural network with an attention mechanism to integrate the shallow high-low frequency features into deep features with high reliability;
(3) Modeling the extracted deep features from both forward and backward directions using a bidirectional long short-term memory network, and performing regression prediction through a fully connected layer.

**Fig 1:**
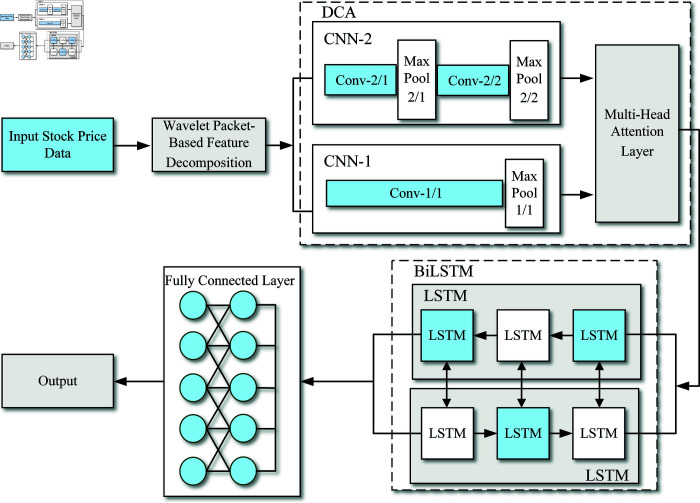
The framework of the stock price prediction method based on the DCA-BiLSTM model.

The specific algorithmic framework is depicted in Fig 1. Below, we elaborate on the three stages involved in constructing this model.

### 2.1 Wavelet packet-based feature decomposition

Stock price data often display marked nonlinearity and non-stationarity, resulting in varied statistical characteristics over time. This challenge complicates the ability of traditional linear models to accurately capture and predict stock price fluctuations. To address this, we employ wavelet packet decomposition, which effectively handles non-stationary time series signals and provides multi-scale information to capture the data’s nonlinear properties [26].

**Fig 2:**
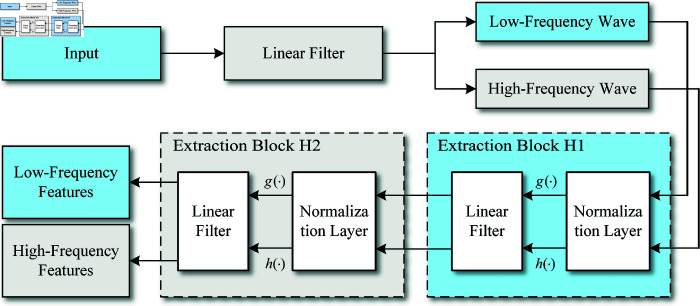
The architecture of the wavelet packet decomposition layer.

Wavelet packet analysis, a signal processing technique based on wavelet transforms, extends the capabilities of traditional methods in terms of time-frequency analysis. While conventional wavelet transforms focus on decomposing signals into approximation and detail components, with a primary emphasis on the low-frequency approximation, wavelet packet analysis provides a more comprehensive decomposition that includes both low- and high-frequency components. This approach extracts features from the entire scale domain, enhancing the scope of feature extraction. In this study, wavelet packet decomposition is applied to initially extract features, decomposing the signals into high- and low-frequency domain information. These features are then further processed by a dual-path convolutional neural network. The architecture of the wavelet packet decomposition layer within the network is illustrated in Fig 2.

As depicted in Fig 2, the wavelet packet decomposition-based shallow feature extraction method uses time series data as input. During processing, multiple high-pass and low-pass linear filtering operations are performed, resulting in the extraction of wavelet packet coefficients from the time series data. These coefficients capture essential signal information and represent the decomposition of signal *x* at scale *j* in the wavelet packet function.

The calculation formula is as follows:

$$\begin{array}{lll} \{\begin{array}{c} x_{j,u}^{2m}=\sqrt{2}\underset{k\in Z}{\sum }g\left(k-2u\right)x_{j-1,k}^{m}\\ x_{j,u}^{2m+1}=\sqrt{2}\underset{k\in Z}{\sum }h\left(k-2u\right)x_{j-1,k}^{m} \end{array}, & \; & \end{array}$$

where $x_{j-1,k}^{m}$ represents the original signal, while $x_{j,u}^{2m}$ and $x_{j,u}^{2m+1}$ denote the decomposed wavelet signals. Here, *m* represents the frequency band, where higher values correspond to higher-frequency bands; *u* represents the time-domain position, *k* indicates the time-domain shift of the filtering function, and *Z* denotes the set of integers. The symbols *g* ( ⋅ )  and *h* ( ⋅ )  represent the high-pass and low-pass filtering functions, respectively, which weight the wavelet packet function values across different frequency bands. Their formulas are as follows:

For the high-pass filtering function, denoted as *g*(*a*):

$$\begin{array}{lll} g(a)=\frac{a_{k}+a_{k+1}}{2}. & \; & \end{array}$$

For the low-pass filtering function, denoted as *h*(*a*):

$$\begin{array}{lll} h(a)=\frac{a_{k}-a_{k+1}}{2}. & \; & \end{array}$$

After passing through the feature extraction layer, the high-frequency time-domain signal $x_{j,u}^{2m}$ and low-frequency time-domain signal $x_{j,u}^{2m+1}$ are transformed into shallow high- and low-frequency features, respectively. While wavelet packet coefficients can effectively reflect the high- and low-frequency features in stock price samples, directly using these coefficients for stock price prediction is challenging [27,28]. The following sections present a model to address the handling of these extracted high- and low-frequency features for more effective application in stock price prediction.

### 2.2 Dual-path convolutional neural network with attention mechanism

Following wavelet packet decomposition, the input data is transformed into high- and low-frequency time-domain features, necessitating further extraction of deeper features. Given the multifaceted impact of factors such as market sentiment, macroeconomic indicators, and company fundamentals on stock prices, a model capable of effectively capturing these relationships is required. To address these challenges, this paper employs a DCA network architecture. This architecture integrates an attention mechanism into a dual-path convolutional neural network, allowing the extraction of highly reliable deep features from high- and low-frequency features via encoder structures with attention mechanisms [29].

The main contributions of this model are as follows:

(1) Due to the differing feature scales of high- and low-frequency signals, optimizing the hyperparameters of different convolutional layers is essential. Therefore, this paper adopts a multi-path convolutional neural network structure;
(2) To address the increased parameter size during convolution, which can prolong training times and reduce model efficiency, global average pooling is used after each convolutional layer to reduce feature dimensionality;
(3) To enhance model accuracy and convergence rate, group normalization is applied to standardize each channel of the feature maps;
(4) In the attention mechanism, scaled dot-product attention is employed to mitigate the complexity and reduce processing speed declines caused by multilinearity.

**Fig 3:**
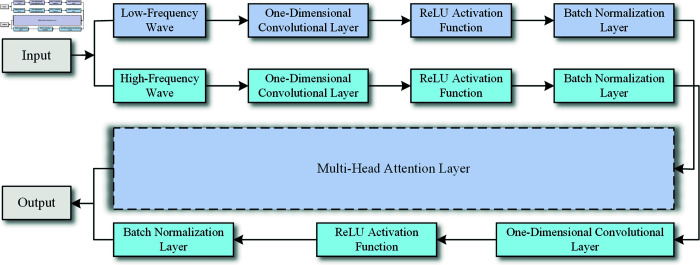
The architecture of the DCA network.

This network architecture, integrating attention mechanisms and dual-channel convolutional networks, aims to process time series inputs more effectively, enhance feature extraction, and support the BiLSTM layer in capturing critical information at various points in sequences. This architecture exhibits impressive deep feature extraction capabilities, as shown in Fig 3.

**Fig 4:**
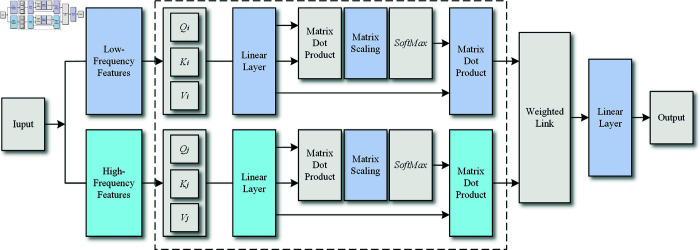
The architecture of the multi-head attention layer.

The multi-head self-attention mechanism includes several self-attention modules, each designed to compute attention weights for every element across positions in the input sequence, resulting in a weighted summation as the output. Illustrated in Fig 4, the architecture unfolds as follows:

(1) First, attention scores are calculated by projecting the input sequence into different spaces using Query $Q_{i}$, Key $K_{i}$, and Value $V_{i}$ matrices. The relevance of each element within the sequence is assessed by computing the attention scores based on the dot product between the query $Q_{i}$ and key *K_i_* pairs.
(2) Next, attention weights are obtained by applying the softmax function to the attention scores, creating a distribution that highlights the significance of each part of the sequence.
(3) A weighted summation is then produced by combining the input sequence with the calculated attention weights.
(4) This weighted sum serves as the output of a single self-attention module.

In a multi-head self-attention mechanism, these steps are repeated independently across multiple attention heads, each with its own parameters. The outputs from each head are then combined, typically by concatenation, to produce the final result, enhancing the model’s ability to capture complex relationships within the sequence.

### 2.3 Bidirectional long short-term memory

Stock prices embody information at different time scales, including short-term fluctuations and long-term trends. Simultaneously considering these scales in prediction is complex. Wavelet packet decomposition aids in modeling data across time scales by providing multi-scale information. BiLSTM, an improvement over LSTM networks, captures both long-term and short-term dependencies, thus addressing time scale issues more comprehensively.

BiLSTM combines forward and backward models, allowing it to capture contextual information bidirectionally [30]. Since a single LSTM only captures context in one direction, using a single LSTM may lead to the loss of features related to the opposite direction, particularly in sequential data like stock prices where context is often bidirectional. Hence, using BiLSTM helps in capturing dependencies more effectively. For stock price time-series data, which are sensitive to time and deeply dependent on past and future periods, BiLSTM improves predictive performance by capturing information across different time scales, including both short-term and long-term trends [31,32].

**Fig 5:**
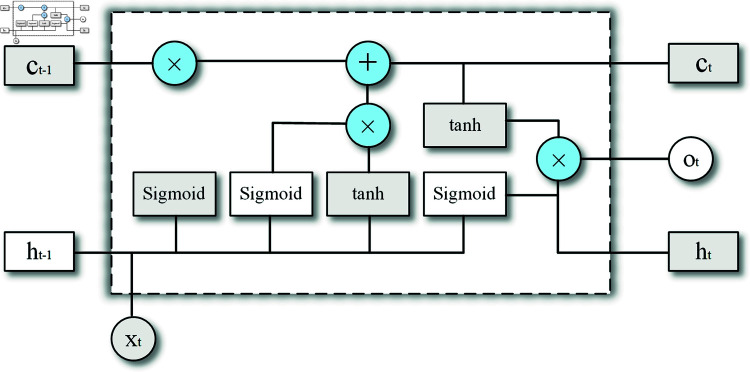
The architecture of a single LSTM unit.

The architecture of a single LSTM unit is illustrated in Fig 5.

The relevant formula definitions are as follows:

$$\begin{array}{llll} i_{t} & =\mathrm{sigmoid}\left(W_{i}\cdot \left[h_{t-1},z_{t}\right]+b_{i}\right),\; & & \;\\ f_{t} & =\mathrm{sigmoid}\left(W_{f}\cdot \left[h_{t-1},z_{t}\right]+b_{f}\right),\; & & \;\\ o_{t} & =\mathrm{sigmoid}\left(W_{o}\cdot \left[h_{t-1},z_{t}\right]+b_{o}\right),\; & & \;\\ {\tilde{c}}_{t} & =\tanh\left(W_{c}\cdot \left[h_{t-1},z_{t}\right]+b_{c}\right),\; & & \;\\ c_{t} & =f_{t}\times c_{t-1}+i_{t}*{\tilde{c}}_{t},\; & & \;\\ h_{t} & =o_{t}\times \tanh\left(c_{t}\right),\; & & \; \end{array}$$

where $z_{t}$ represents the input data at the current time step *t*. $i_{t}$ represents the input gate, controlling information flow into the memory cell $c_{t}$. $f_{t}$ is the forget gate, deciding whether information from the previous time step *t*–1 should be retained in the memory cell $c_{t}$. $o_{t}$ determines whether the memory cell information impacts the current hidden state $h_{t}$. $c_{t}$ denotes the memory cell, embodying the neural state’s memory, and giving LSTM units the capacity to store, read, reset, and update historical information. The $W_{*}$ are weights associated with respective gates. Sigmoid and tanh are the commonly used activation functions [33].

## 3 Experiments

To assess the performance of the proposed stock price prediction model based on DCA-BiLSTM, datasets from four sources were selected: Apple stock, Google stock, Tesla stock, and the Nasdaq index data. The proposed model was compared with four alternative models: CNN-BiLSTM, DCA, CNN, and BiLSTM [34–36].

### 3.1 Experimental environment and parameters

The experiments were conducted in a Windows 10 22H2 environment. Python 3 and the PyTorch neural network framework were used to implement the DCA-BiLSTM model. CUDA 11.8 was employed to accelerate model training.

### 3.2 Experimental datasets

The datasets for this study, sourced from Yahoo Finance (https://finance.yahoo.com/), contain historical daily price data for Apple, Google, Tesla stocks, and the Nasdaq index, with all data collection and usage complying with Yahoo Finance’s Terms of Service. Apple, as a global leader in technology, serves as a key market indicator. Google, one of the largest players in the internet industry, impacts the advertising sector and the broader IT field. Tesla, a pioneer in electric vehicles and renewable energy, offers insights into emerging markets, while the Nasdaq index, representing a range of technology companies, provides a broad view of technology sector trends.

**Fig 6:**
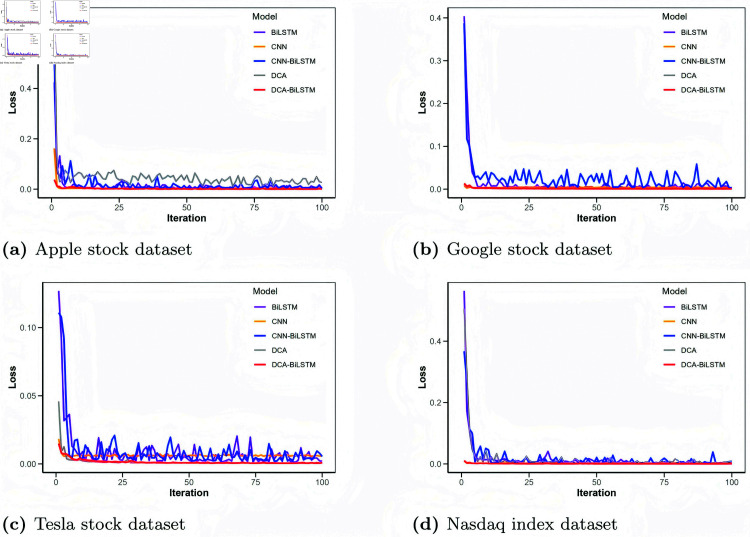
Comparison of loss values for different models.

**Fig 7:**
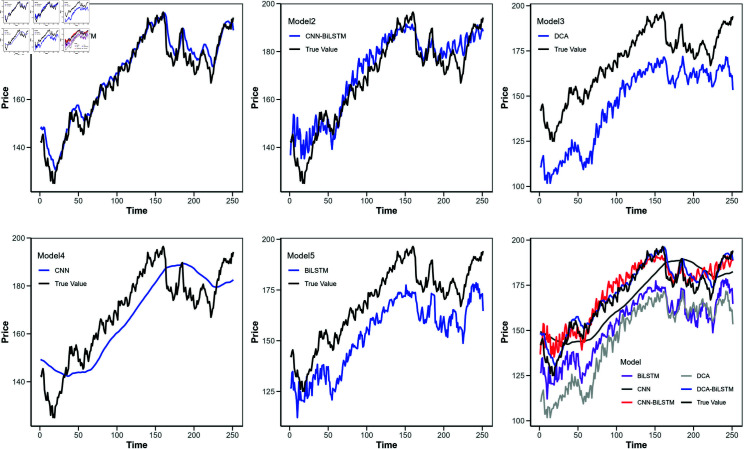
Comparison of predicted values for different models (Apple stock).

The study spans the following timeframes: Apple stock (2013/12/9-2023/12/7), Google stock (2014/1/13-2024/1/12), Tesla stock (2014/1/13-2024/1/12), and the Nasdaq index (2013/12/9-2023/12/7). The selected features include indicators such as opening price, highest price, lowest price, price change, percentage change, trading volume, turnover, market capitalization, and total market value, among others. These datasets comprise a diverse range of fundamental data, supporting the construction of multifactorial time-series samples and enabling comprehensive predictive analysis. The experimental models primarily utilize closing prices to forecast future market trends.

### 3.3 Evaluation metrics

In the experiment, we utilize four metrics to assess the predictive performance of the model, namely the coefficient of determination ($R^{2}$), mean absolute error (MAE), mean absolute percentage error (MAPE), and root mean squared error (RMSE). The formulas for calculating these metrics are as follows:

$$\begin{array}{llll} R^{2} & =1-\frac{\overset{n_{test}}{\underset{i=1}{\sum }}{\left(N_{test,i}-{\hat{N}}_{test,i}\right)}^{2}}{\overset{n_{test}}{\underset{i=1}{\sum }}{\left({\bar{N}}_{test}-N_{test,i}\right)}^{2}},\; & & \;\\ MAE & =\frac{1}{n_{test}}\overset{n_{test}}{\underset{i=1}{\sum }}\left|N_{test,i}-{\hat{N}}_{test,i}\right|,\; & & \;\\ MAPE & =\frac{1}{n_{test}}\overset{n_{test}}{\underset{i=1}{\sum }}\left|\frac{N_{test,i}-{\hat{N}}_{test,i}}{N_{test,i}}\right|,\; & & \;\\ RMSE & =\sqrt{\frac{1}{n_{test}}\overset{n_{test}}{\underset{i=1}{\sum }}{\left(N_{test,i}-{\hat{N}}_{test,i}\right)}^{2}},\; & & \; \end{array}$$

where $n_{test}$ represents the sample size of the test set, and $N_{test,i}$, ${\bar{N}}_{test}$, and ${\hat{N}}_{test,i}$ denote the true values, the mean of all true values in the test set, and the predicted values of the test set, respectively. These four metrics individually quantify the disparities between the model’s predicted values and the actual observations. $R^{2}$ indicates the model’s explanatory power, while MAE, MAPE, and RMSE quantify the magnitude of prediction errors. Higher $R^{2}$ values (closer to 1) and lower MAE, MAPE, and RMSE values generally indicate superior predictive performance.

**Fig 8:**
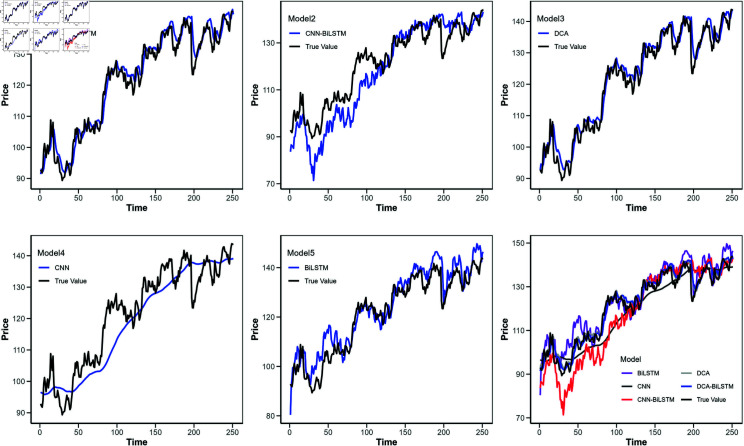
Comparison of predicted values for different models (Google stock).

**Fig 9:**
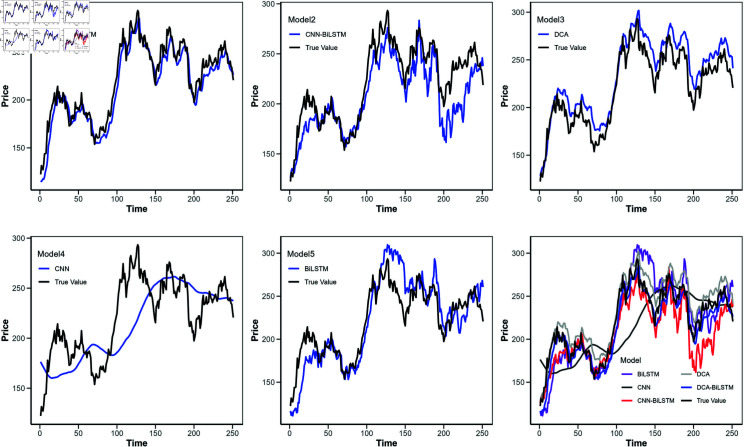
Comparison of predicted values for different models (Tesla stock).

**Fig 10:**
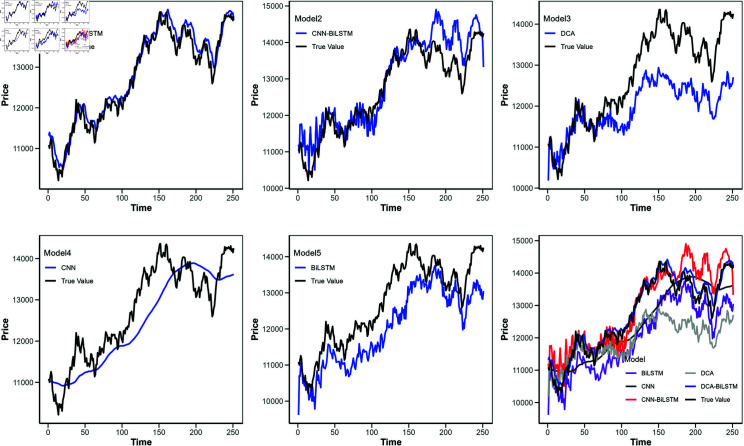
Comparison of predicted values for different models (Nasdaq index).

**Table 1:** Comparison of evaluation metrics for different models.

Datasets	Models	$R^{2}$	MAE	MAPE	RMSE
Apple stock	DCA-BiLSTM	0.9507	3.5970	0.0192	4.1158
	CNN-BiLSTM	0.8868	4.8763	0.0305	6.2350
	DCA	0.6996	9.3914	0.0566	10.1557
	CNN	0.1905	15.9490	0.1401	24.1788
	BiLSTM	0.3356	13.5497	0.0944	16.6720
Google stock	DCA-BiLSTM	0.9595	2.4250	0.0207	3.1841
	CNN-BiLSTM	0.7427	6.2594	0.0554	8.0303
	DCA	0.9567	2.5022	0.0212	3.2921
	CNN	0.8418	4.9632	0.0417	6.2955
	BiLSTM	0.8796	4.3224	0.0374	5.4932
Tesla stock	DCA-BiLSTM	0.9077	9.1389	0.0428	11.4288
	CNN-BiLSTM	0.6614	17.0838	0.0752	21.8854
	DCA	0.8111	14.0728	0.0650	16.3476
	CNN	0.3350	23.6805	0.1098	30.6716
	BiLSTM	0.6498	16.9560	0.0777	22.2567
Nasdaq index	DCA-BiLSTM	0.9594	183.0246	0.0146	230.0247
	CNN-BiLSTM	0.8064	396.4562	0.0314	502.0048
	DCA	0.7276	498.9665	0.0388	595.5876
	CNN	0.2620	828.9291	0.0623	980.2409
	BiLSTM	0.5169	700.4817	0.0544	793.1271

### 3.4 Experimental results

To validate the proposed DCA-BiLSTM model, we compared it against four alternative models: CNN-BiLSTM, DCA, CNN, and BiLSTM. To enhance the predictive precision of the model, we allocated 90% of the dataset for training to refine parameter tuning, while the remaining 10% was reserved for testing to assess the model’s performance.

Fig 6 shows the loss function curves of these models, with the horizontal axis indicating the iteration count and the vertical axis representing the loss value. Figs 7 to 10 illustrate the closing price predictions of these models, with the horizontal axis representing the number of iterations and the vertical axis representing the closing price.

In Fig 6, the proposed DCA-BiLSTM model demonstrates relatively lower initial loss values across all datasets compared to the other models. With increasing iterations, all models exhibit a gradual reduction in loss values; however, the DCA-BiLSTM model maintains a more stable loss curve, highlighting its robustness and ability to achieve consistently lower loss levels across datasets.

Observing Figs 7 to 10 reveals that in the early iterations, the DCA-BiLSTM model trends closer to actual closing prices than the other models. As iterations increase, this proximity becomes more pronounced, while the predictions of the other models display larger deviations.

Table 1 presents the predictive results for each dataset.

The analysis of predictive performance across the Apple, Google, Tesla, and Nasdaq datasets reveals that the DCA-BiLSTM model consistently outperforms other models. For Apple stock, it achieves an $R^{2}$ of 0.9507, with superior accuracy across MAE, MAPE, and RMSE metrics. On the Google stock dataset, the model stands out with an $R^{2}$ of 0.9595 and the lowest MAE, RMSE, and MAPE, demonstrating precise price predictions. Similarly, for Tesla stock, the DCA-BiLSTM model achieves the highest $R^{2}$ at 0.9077, indicating strong predictive performance across all metrics. Finally, on the Nasdaq index, the model attains an $R^{2}$ of 0.9594 and outperforms in error metrics, underscoring its robustness and accuracy across varied stock datasets.

## 4 Conclusion and future work

This paper proposes a stock price prediction model based on the DCA-BiLSTM approach. The method begins with decomposing the time-series data using wavelet packet decomposition to extract high- and low-frequency features. Subsequently, a neural network architecture integrating convolutional layers and attention mechanisms performs deep feature extraction. A bidirectional LSTM model is then applied to capture forward and backward context within the data. Experimental results demonstrate the DCA-BiLSTM model’s accuracy and stability in stock price prediction, underscoring its capacity to capture complex temporal relationships and improve prediction precision.

Nonetheless, several challenges remain for future research. First, stock markets are influenced by numerous external factors such as political events, natural disasters, and economic policies, which existing models may inadequately address. Future studies should explore integrating these factors to enhance model robustness and applicability. Additionally, although our model performs well in short-term predictions, its accuracy and stability diminish in long-term forecasting due to factors such as economic cycles and industry trends that are difficult to capture. Therefore, future research should focus on addressing long-term prediction challenges through specialized modeling techniques. Lastly, current models face overfitting risks, especially when handling large datasets. Future studies should explore strategies to prevent overfitting and improve model generalization and robustness.

In conclusion, while this study has achieved promising results, stock price prediction remains a complex and evolving field that demands continuous exploration and refinement of predictive techniques. Future research will continue to investigate innovative methodologies to better capture stock market patterns, providing investors with more accurate and reliable forecasting insights.
